# Linkage analysis of longitudinal data and design consideration

**DOI:** 10.1186/1471-2156-7-37

**Published:** 2006-06-12

**Authors:** Heping Zhang, Xiaoyun Zhong

**Affiliations:** 1 Department of Epidemiology and Public Health, Yale University School of Medicine, 60 College Street, New Haven, CT 06520-8034, USA; 2 Department of Medicine, University of Massachusetts Medical School, 55 Lake Avenue North, Worcester, MA 01655, USA

## Abstract

**Background:**

Statistical methods have been proposed recently to analyze longitudinal data in genetic studies. So far, little attention has been paid to examine the relationship among key factors in genetic longitudinal studies including power, the number of families or sibships, and the number of repeated measures per individual subjects.

**Results:**

We proposed a variance component model that extends classic variance component models for a single quantitative trait to mapping longitudinal traits. Our model includes covariate effects and allows genetic effects to vary over time. Using our proposed model, we examined the power, pedigree structures, and sample size through simulation experiments.

**Conclusion:**

Our simulation results provide useful insights into the study design for genetic, longitudinal studies. For example, collecting a small number of large sibships is much more powerful than collecting a large number of small sibships or increasing the number of repeated measures, when the total number of measurements is comparable.

## Background

Longitudinal study design has been routinely used to investigate the etiology and epidemiology of complex diseases, and statistical methods for analyzing longitudinal data are well established [1]. However, there are limited applications of longitudinal data in genetic studies.

Province and Rao [2] used path analysis for assessing familial aggregation in the presence of temporal trends, although their analysis did not include genetic marker information. Longitudinal studies have also been used in a few occasions for twin and adoption studies (e.g., [3-6]). However, the main purpose of those studies was to assess the heritability of a trait, instead of mapping candidate loci.

Using an ad hoc approach, Levy and colleagues [7] conducted a linkage scan of the Framingham Heart Study. They regress the phenotype against covariates as in a standard mixed effects model, and then treat the residuals corresponding to individual measurements as a quantitative trait in standard linkage analysis software such as SOLAR [8]. More recently, in the Genetic Analysis Workshop 13, some participants examined two-step models and some proposed joint models [9]. The first step in a two-step model is similar to that of Levy et al. [7] by fitting an "ordinary" longitudinal model without consideration for genetic markers or family structures. Then, in the second step, linkage analysis is performed on one or more statistics derived from the first step. While such two-step methods are practical and simple, they are not ideal. For example, even if the covariates have additive effects to the genetic effects, potential useful information can be lost in deriving the residuals or some summary statistics. Besides, the selection among different statistics (e.g., residuals and averages) to be used in the second stage increases the number of tests to be performed, which raises the multiple comparison issue. Also importantly, the lack of a well-defined statistical model directly associating the original phenotype to the inheritance of the markers makes it infeasible to conduct formal statistical inference. In fact, the authors in the Genetic Analysis Workshop 13 [9] clearly pointed out that a joint approach to simultaneously estimating genetic and longitudinal model parameters is appealing, because estimates of genetic and longitudinal parameters will be mutually adjusted for one another. Thus, in this report, we consider a joint model that is related to some of the models described in [9]. Our main objective is to use our model to examine the relationship among key factors in genetic longitudinal studies including power, the number of families or sibships, and the number of repeated measures per individual subjects.

There is a growing effort to develop mixed effects models that separate the genetic effect from environmental effects [10] and that incorporate temporal information [11]. However, those models do not have simple structures to accommodate genetic and temporal interactions, or to enable us to assess the longitudinal study design in linkage analysis. This raises the computational concern and may limit the analyses that can be performed as pointed in [9]. Hence, our idea is to use a realistic yet simple variance component model that can be used to analyze general pedigree data such as the Framingham Heart Study and that allows us to consider age specific genetic effects and related study design issues. We choose a variance component model because this type of models is well established for linkage analysis of quantitative traits (e.g., [8,12,13]).

## Results

In this section, we report our simulation results to assess the Type I error rate based on the asymptotic theory, and the power of our method in detecting linkage. We are particularly interested in the effectiveness of repeated measures in improving the power. For example, how do we determine the most cost-effective number of repeated measures? The computation was performed by a statistical software R using our own program, which are available upon request. We should note that our model and program have been used to analyze general pedigree data such as the Framingham Heart Study (to be reported in a future report), although our simulation below is focused on sibships to reduce computational burden. Nuclear families were simulated, and fully informative markers with four equally frequent alleles were generated. All parental alleles were distinguished. For the nuclear families, phenotypes were simulated only for the siblings. In all the simulations, each sib in every nuclear family has 5 measurements taken at different times. The measurement times were simulated simply as (1, 2, 3, 4, 5). A covariate was simulated from a uniform distribution between 0 and 1. For clarity, we used *f*(*X*, *t*) = *Xβ *to generate the data, where *β *= (*β*_0_, *β*_1_, *β*_2_)' = (1, 1, 1)' and *β*_0_, *β*_1 _and *β*_2 _are parameters for the intercept, the time and the simulated covariate in mean structure. As in related studies [13], we did not consider dominant effects in the simulation studies and set ($\sigma_{d1}^{2}$, $\sigma_{d2}^{2}$) = (0, 0).

### Type I error rates

To evaluate the type 1 error rates of the proposed tests, we considered two different null models. The first type of null model assumes that the genetic linkage effect due to the testing QTL and the polygenic effect are both zero, that is, ($\sigma_{a1}^{2}$, $\sigma_{a2}^{2}$) = (0, 0). The second type of null model assumes there is no genetic linkage effect due to the testing QTL but there is some polygenic effect and ($\sigma_{a1}^{2}$, $\sigma_{a2}^{2}$) = (0, 1). We also simulated a measurement error from a normal distribution with the variance *σ*^2 ^equal to 7 and the autocorrelation between measurements at two time points *t *and *u *for a sib equals exp(-0.5|*t *- *u|*). We considered in the analysis two choices of *s*(*t*): linear [*s*(*t*) = *s*_0 _+ *s*_1_*t*] and quadratic [*s*(*t*) = *s*_0 _+ *s*_1_*t *+ *s*_2_*t*^2^]. We simulated 5,000 replications of 100 sib pairs.

Likelihood ratio test is used to test the null hypothesis that the genetic variance due to the testing QTL equals zero (no linkage).

We use two times the natural logarithm of the likelihood ratio as the test statistic. Its asymptotic distribution appears to be a mixture of *χ*^2 ^distributions [16], but the degrees of freedom depend on *s*(*t*).

When *s*(*t*) is constant, the model is equivalent to the traditional variance-component model since we can consider only one independent parameter, i.e., either *s*_0 _or $\sigma_{a1}^{2}$. In this case, the test statistic asymptotically follows $\frac{1}{2}\chi_{0}^{2}+\frac{1}{2}\chi_{1}^{2}$. For a linear *s*(*t*), the asymptotic distribution of the test statistic appears to be $\frac{1}{2}\chi_{1}^{2}+\frac{1}{2}\chi_{2}^{2}$. For a quadratic *s*(*t*), the asymptotic distribution of the test statistic appears to be $\frac{1}{2}\chi_{2}^{2}+\frac{1}{2}\chi_{3}^{2}$. Because we do not have theoretical proofs for the asymptotic distributions of the test statistic, we derived critical values empirically through simulations.

In practice, we do not know the form of *s*(*t*). However, we can use the backward selection as in regression analysis by beginning with the quadratic polynomial and testing whether the coefficients are zero or not. This strategy can serve as the guide in determining the final form of *s*(*t*).

Table 1 presents the empirical type I error rates based on 5,000 simulated replications under two null models. The rejection rates in the table were obtained by computing the frequencies at which the null hypotheses were rejected at the critical values from the stated asymptotic distributions. Given that we used only 100 sib pairs, the empirical type I error rates are numerically close to the nominal significance levels.

**Table 1:** Type 1 error rate comparisons based on 5000 simulations of 100 sib pairs under two different types of null models. Null model A is the model simulated under no heritability due to the testing QTL and no polygenic heritability. Null model B is the model without heritability due to the testing QTL but with polygenic heritability *h*^2 ^and $\sigma_{a2}^{2}$ = 1. The other underlying parameters are (*β*_0_, *β*_1_, *β*_2_) = (1, 1, 1), and (*σ*^2^, *α*) = (7, 0.5). The assumed *s*(*t*) is labeled as "i" for *s*_0 _+ *s*_1_*t*, and "q" for *s*_0 _+ *s*_1_*t *+ *s*_2_*t*^2^.

*T*	*h*^2^	Assumed *s*(*t*)	Significance level		
			
			0.05	0.01	0.001
Null model A					
5	0.0	l	0.045	0.010	0.0012
		q	0.051	0.011	0.0008
4	0.0	l	0.041	0.009	0.0005
		q	0.048	0.009	0.0005
2	0.0	l	0.042	0.005	0.0008
		q	0.040	0.006	0.0005

Null model B					
5	0.190	l	0.040	0.006	0.0006
		q	0.053	0.009	0.0014
4	0.174	l	0.041	0.008	0.0004
		q	0.052	0.010	0.0006
2	0.141	l	0.040	0.008	0.0008
		q	0.038	0.005	0.0006

### Power comparisons

To compare the power increment from larger sibships, we considered the scenarios of collecting 200 sib pairs, 400 sib pairs and 200 nuclear families with 4 siblings each so that we can assess the corresponding effects of the number of nuclear families, the size of the nuclear families, and the number of repeated measures on power. We simulated data from the following three forms of *s*(*t*): (a) *s*(*t*) = 1 + 0.1*t*; (b) *s*(*t*) = 1; (c) $s(t)=\frac{\exp(t)}{1+\exp(t)}$. We also generated measurement errors from a multivariate normal distribution with the variance *σ*^2 ^and the within-subject autocorrelation exp(-*α|t - u|*) between measurements at two time points *t *and *u*. To evaluate the power, we conducted a number of experiments using various genetic models: (a) ($\sigma_{a1}^{2}$, $\sigma_{a2}^{2}$, *σ*^2^, *α*)' = (2, 1, 7, 0.5)'; (b) ($\sigma_{a1}^{2}$, $\sigma_{a2}^{2}$, *σ*^2^, *α*)' = (1, 1, 8, 0.5)'; (c) ($\sigma_{a1}^{2}$, $\sigma_{a2}^{2}$, *σ*^2^, *α*)' = (0.5, 1, 8.5, 0.5)'. Note that these four parameters determine the extent of the overall genetic heritability as well as the heritability due to a specific locus under consideration.

When presenting our power assessment, we make use of a generalized heritability measure for longitudinal trait proposed by de Andrade et al. [11]. To incorporate the serial variance components, we express the polygenic and major gene heritabilities in our model as

$\begin{array}{c} h^{2}=\frac{\sum_{i}\frac{T_{i}(T_{i}+1)}{2}\sigma_{a2}^{2}}{\sum_{i}\left\{\sum_{t=1}^{T_{i}}s{\left(t\right)}^{2}\sigma_{a1}^{2}+\frac{T_{i}(T_{i}+1)}{2}\sigma_{a2}^{2}+T_{i}\sigma^{2}+\sum_{1\leq t<t^{\prime }\leq T_{i}}\left[s(t)s(t^{\prime })\sigma_{a1}^{2}+\sigma^{2}\exp(-\alpha |t-t^{\prime }|)\right]\right\}},\\ h_{g}^{2}=\frac{\sum_{i}[\sum_{t=1}^{T_{i}}s{\left(t\right)}^{2}+\sum_{1\leq t<t^{\prime }\leq T_{i}}s(t)s(t^{\prime })]\sigma_{a1}^{2}}{\sum_{i}\left\{\sum_{t=1}^{T_{i}}s{\left(t\right)}^{2}\sigma_{a1}^{2}+\frac{T_{i}(T_{i}+1)}{2}\sigma_{a2}^{2}+T_{i}\sigma^{2}+\sum_{1\leq t<t^{\prime }\leq T_{i}}\left[s(t)s(t^{\prime })\sigma_{a1}^{2}+\sigma^{2}\exp(-\alpha |t-t^{\prime }|)\right]\right\}}. \end{array}$

Table 2 displays the polygenic and major gene heritabilities used in our simulation models when different numbers of repeated measurements are used.

**Table 2:** The polygenic and major gene heritabilities (*h*^2 ^and $h_{g}^{2}$) used in our simulation models

	($\sigma_{a1}^{2}$, $\sigma_{a2}^{2}$, σ^2^, α)					
	
	(2, 1, 7, 0.5)		(1, 1, 8, 0.5)		(0.5, 1, 8.5, 0.5)	
			
*T*	*h*^2^	$h_{g}^{2}$	*h*^2^	$h_{g}^{2}$	*h*^2^	$h_{g}^{2}$
*s*(*t*) = 1 + 0.1*t*						
2	0.102	0.272	0.108	0.143	0.111	0.073
3	0.108	0.312	0.117	0.169	0.122	0.088
4	0.113	0.353	0.125	0.196	0.133	0.104
5	0.116	0.392	0.132	0.224	0.143	0.121

*s*(*t*) = 1						
2	0.110	0.220	0.112	0.112	0.113	0.056
3	0.120	0.240	0.123	0.123	0.125	0.063
4	0.129	0.258	0.135	0.135	0.138	0.069
5	0.138	0.276	0.146	0.146	0.150	0.075

$s(t)=\frac{\exp(t)}{\left(1+\exp(t)\right)}$						
2	0.119	0.155	0.116	0.076	0.115	0.037
3	0.128	0.188	0.128	0.093	0.127	0.047
4	0.137	0.215	0.139	0.109	0.140	0.055
5	0.145	0.239	0.150	0.124	0.152	0.063

traditional VC	0.1	0.2	0.1	0.1	0.1	0.05

Regardless of the true form of *s*(*t*), in our estimation we assumed *s*(*t*) to be one of the following three forms: *s*(*t*) = *s*_0_, *s*(*t*)*= s*_0 _+ *s*_1_*t*, and *s*(*t*)* = s*_0 _+ *s*_1_*t *+ *s*_2_*t*^2 ^where *s*_0 _is nonnegative, and it may need to be estimated together with s_1 _and/or *s*_2_, depending on the choice. As stated above, one of the true *s*(*t*)'s is the logit function. This is because we want to know what happens in linkage detection when *s*(*t*) is misspecified.

To understand the gain of power as a result of more repeated measures, we examined the power using all or some of the 5 measurements for each sib. We also compared the power from our models with the power of using traditional variance component (VC) method for a single measurement. The single measure can be a measurement at a particular time point or the average of the five measurements for each sib.

Tables 3, 4, and 5 display the power in the experiments as specified above. To appreciate the incremental gain of power as the number of repeated measures increases, we compared the power estimates when we used all or some of the 5 repeated measurements. As expected, the power increases as the number of repeated measures and/or the number of families increase. However, the increment of power is not uniform, and depends on the significance level. For example, ascertaining 200 sib pairs with four repeated measures tends to yield better power than collecting 400 sib pairs with two repeated measures when there is a gene-time interaction, and vice versa when there is no gene-time interaction. The information from these tables underscores the importance to conduct the power calculation under the specific designs and significance level in order to choose the most cost effective designs.

**Table 3:** The power comparisons based on 500 replicates. The underlying parameters are (*β*_0_, *β*_1_, *β*_2_) = (1, 1, 1), and ($\sigma_{a1}^{2}$, $\sigma_{a2}^{2}$, *σ*^2^, *α*) = (2, 1, 7, 0.5). The assumed *s*(*t*) is labeled as "c" for constant, "l" for *s*_0 _+ *s*_1_*t*, and "q" for *s*_0 _+ *s*_1_*t *+ *s*_2_*t*^2^.

			200 sib pairs				400 sib pairs			
				
*T*	$h_{g}^{2}$	Assumed *s*(*t*)	Significance level				Significance level			
				
			0.05	0.01	0.001	0.0001	0.05	0.01	0.001	0.0001
True *s*(*t*) = 1 + 0.1*t*										
5	0.392	c	0.77	0.56	0.26	0.09	0.97	0.84	0.62	0.36
		l	0.90	0.78	0.45	0.24	0.99	0.95	0.86	0.74
		q	0.86	0.64	0.39	0.19	0.99	0.95	0.84	0.67
4	0.353	c	0.68	0.42	0.16	0.04	0.89	0.72	0.44	0.23
		l	0.75	0.52	0.23	0.07	0.95	0.87	0.65	0.47
		q	0.66	0.42	0.18	0.04	0.93	0.81	0.59	0.38
2	0.272	c	0.43	0.20	0.04	0.01	0.63	0.40	0.19	0.06
		l	0.34	0.16	0.04	0.00	0.59	0.37	0.13	0.04
		q	0.20	0.06	0.02	0.00	0.42	0.23	0.07	0.02
5	0.2	*	0.77	0.54	0.2	0.06	0.97	0.83	0.60	0.34
1	0.2	**	0.33	0.13	0.03	0.01	0.60	0.35	0.06	0.00

True *s*(*t*) = 1										
5	0.276	c	0.49	0.25	0.07	0.01	0.74	0.48	0.20	0.06
		l	0.33	0.15	0.02	0.00	0.60	0.33	0.10	0.03
		q	0.19	0.07	0.01	0.00	0.51	0.25	0.09	0.02
4	0.258	c	0.45	0.21	0.05	0.01	0.67	0.42	0.15	0.03
		l	0.27	0.11	0.02	0.00	0.60	0.33	0.09	0.01
		q	0.16	0.05	0.01	0.00	0.51	0.25	0.05	0.01
2	0.220	c	0.35	0.12	0.02	0.00	0.50	0.24	0.03	0.01
		l	0.17	0.03	0.01	0.00	0.32	0.14	0.01	0.01
		q	0.08	0.01	0.01	0.00	0.18	0.05	0.01	0.01
5	0.2	*	0.46	0.19	0.04	0.01	0.71	0.46	0.19	0.05
1	0.2	**	0.25	0.07	0.01	0.00	0.46	0.17	0.03	0.01

True *s*(*t*) = exp(*t*)/(1 + exp(*t*))										
5	0.239	c	0.40	0.15	0.02	0.00	0.59	0.36	0.15	0.03
		l	0.42	0.22	0.04	0.00	0.62	0.41	0.14	0.06
		q	0.35	0.17	0.03	0.00	0.69	0.48	0.18	0.05
4	0.215	c	0.35	0.15	0.02	0.00	0.51	0.25	0.06	0.01
		l	0.37	0.16	0.03	0.01	0.61	0.32	0.11	0.03
		q	0.24	0.10	0.02	0.00	0.55	0.31	0.11	0.02
2	0.155	c	0.20	0.04	0.01	0.00	0.33	0.14	0.02	0.00
		l	0.22	0.07	0.00	0.00	0.35	0.14	0.03	0.01
		q	0.09	0.02	0.00	0.00	0.21	0.06	0.02	0.01
5	0.2	*	0.40	0.14	0.03	0.00	0.52	0.31	0.11	0.03
1	0.2	**	0.15	0.02	0.01	0.00	0.22	0.10	0.00	0.00

*based on the average measurement; ** based on one measurement.

**Table 4:** The power comparisons based on 500 replicates. The underlying parameters are (*β*_0_, *β*_1_, *β*_2_) = (1, 1, 1), and ($\sigma_{a1}^{2}$, $\sigma_{a2}^{2}$, *σ*^2^, *α*) = (1, 1, 8, 0.5). The assumed *s*(*t*) is labeled as "c" for constant, "l" for *s*_0 _+ *s*_1_*t*, and "q" for *s*_0 _+ *s*_1_*t *+ *s*_2_*t*^2^.

			200 sib pairs				400 sib pairs			
				
*T*	$h_{g}^{2}$	Assumed *s*(*t*)	Significance level				Significance level			
				
			0.05	0.01	0.001	0.0001	0.05	0.01	0.001	0.0001
True *s*(*t*) = 1 + 0. 1*t*										
5	0.224	c	0.35	0.12	0.02	0.00	0.59	0.34	0.12	0.02
		l	0.41	0.17	0.05	0.01	0.70	0.45	0.19	0.07
		q	0.35	0.13	0.03	0.004	0.63	0.38	0.14	0.05
4	0.196	c	0.28	0.09	0.01	0.00	0.47	0.22	0.05	0.01
		l	0.30	0.10	0.02	0.00	0.51	0.28	0.06	0.01
		q	0.25	0.05	0.02	0.004	0.44	0.24	0.05	0.02
2	0.143	c	0.15	0.02	0.00	0.00	0.30	0.09	0.00	0.00
		l	0.12	0.032	0.00	0.00	0.21	0.07	0.01	0.00
		q	0.06	0.004	0.00	0.00	0.09	0.02	0.00	0.00
5	0.1	*	0.32	0.11	0.02	0.00	0.53	0.28	0.06	0.01
1	0.1	**	0.11	0.01	0.00	0.00	0.24	0.07	0.00	0.00

True *s*(*t*) = 1										
5	0.146	c	0.16	0.04	0.01	0.00	0.28	0.12	0.02	0.00
		l	0.11	0.04	0.01	0.00	0.22	0.07	0.01	0.00
		q	0.10	0.02	0.00	0.00	0.18	0.06	0.00	0.00
4	0.135	c	0.13	0.03	0.0	0.00	0.28	0.07	0.02	0.00
		l	0.09	0.02	0.00	0.00	0.20	0.05	0.00	0.00
		q	0.08	0.02	0.00	0.00	0.15	0.03	0.01	0.00
2	0.112	c	0.11	0.02	0.00	0.00	0.19	0.04	0.00	0.00
		l	0.09	0.02	0.00	0.00	0.14	0.02	0.00	0.00
		q	0.06	0.01	0.00	0.00	0.06	0.00	0.00	0.00
5	0.1	*	0.15	0.03	0.01	0.00	0.26	0.09	0.01	0.01
1	0.1	**	0.06	0.02	0.00	0.00	0.13	0.01	0.00	0.00

True *s*(*t*) = exp(*t*)/(1 + exp(*t*))										
5	0.124	c	0.15	0.04	0.00	0.00	0.27	0.08	0.014	0.00
		l	0.15	0.05	0.01	0.00	0.25	0.10	0.020	0.00
		q	0.15	0.03	0.00	0.00	0.20	0.07	0.014	0.00
4	0.109	c	0.13	0.03	0.00	0.00	0.19	0.05	0.00	0.00
		l	0.12	0.04	0.01	0.00	0.19	0.08	0.01	0.00
		q	0.11	0.02	0.01	0.00	0.18	0.06	0.01	0.00
2	0.076	c	0.08	0.02	0.00	0.00	0.14	0.03	0.00	0.00
		l	0.08	0.01	0.01	0.00	0.10	0.02	0.01	0.01
		q	0.03	0.01	0.00	0.00	0.06	0.01	0.01	0.00
5	0.1	*	0.14	0.03	0.01	0.00	0.26	0.06	0.01	0.00
1	0.1	**	0.06	0.01	0.00	0.00	0.10	0.01	0.00	0.00

*based on the average measurement; ** based on one measurement.

**Table 5:** The power based on 500 replicates of 200 4-sib families. For comparison purpose with the other tables, we consider two repeated measures only. The underlying parameters are (*β*_0_, *β*_1_, *β*_2_) = (1, 1, 1) with various settings of ($\sigma_{a1}^{2}$, $\sigma_{a2}^{2}$, *σ*^2^, *α*). The assumed *s*(*t*) is labeled as "c" for constant, "l" for *s*_0 _+ *s*_1_*t*, and "q" for *s*_0 _+ *s*_1_*t *+ *s*_2_*t*^2^.

($\sigma_{a1}^{2}$, $\sigma_{a2}^{2}$, *σ*^2^, α)	$h_{g}^{2}$	Assumed *s*(*t*)	Significance level			
			
			0.05	0.01	0.001	0.0001
True *s*(*t*) = 1 + 0.1*t*						
(2, 1, 7, 0.5)	0.272	c	0.98	0.93	0.77	0.52
		l	0.97	0.85	0.71	0.47
		q	0.89	0.79	0.56	0.35

(1, 1, 8, 0.5)	0.143	c	0.57	0.35	0.11	0.04
		l	0.48	0.26	0.10	0.04
		q	0.33	0.16	0.05	0.00

True *s*(*t*) = 1						
(2, 1, 7, 0.5)	0.220	c	0.91	0.77	0.49	0.25
		l	0.84	0.65	0.38	0.13
		q	0.72	0.51	0.22	0.08

(1, 1, 8, 0.5)	0.112	c	0.44	0.24	0.06	0.01
		l	0.37	0.12	0.03	0.00
		q	0.22	0.07	0.01	0.00

True *s*(*t*) = exp(*t*)/(1 + exp(*t*))						
(2, 1, 7, 0.5)	0.155	c	0.66	0.40	0.17	0.06
		l	0.65	0.44	0.19	0.07
		q	0.53	0.31	0.10	0.05

(1, 1, 8, 0.5)	0.076	c	0.28	0.11	0.02	0.00
		l	0.25	0.07	0.01	0.00
		q	0.11	0.02	0.01	0.00

Tables 3 and 4 reveal serious loss of power of ignoring a gene-time interaction. For example, in Table 3 when the underlying *s*(*t*) = 1 + 0.1*t*, with 5 repeated measures, the power estimates by ignoring *s*(*t*) were 0.77, 0.56, 0.26, and 0.09, respectively, at significance levels 0.05, 0.01, 0.001, and 0.0001. In contrast, the respective power estimates were increased to 0.90, 0.78, 0.45, and 0.24 when we estimated *s*(*t*) from *s*_0 _+ s_1_*t*. We should also note here that the fold of increase is more dramatic for a more stringent significance level. On the other hand, is there a loss of the power if we consider *s*(*t*) when there is no time-dependent genetic effort? Or, broadly, what happens to the power if the time-dependent effect is misspecified? Tables 3, 4, and 5 address these questions. As expected, the power is at its peak when the underlying time trend is correctly specified. However, even with a misspecified trend, the test based on our model is more powerful than the one using a single measure, regardless of whether it was from a particular age or the average of the same number of repeated measures. We should note that, from our experiment, the use of the average of repeated measures yields more power than the use of a single measure at a given time point. In other words, without any consideration for the cost and effectiveness, we gain power from repeated measures even with a simple approach.

Finally, Table 3, 4, and 5 reveal the substantial benefit of power as a result of ascertaining large pedigrees. Table 5 displays the power of using 200 4-siblings. The power estimates using 400 sib pairs is available in Tables 3 and 4. Clearly, whenever feasible, collecting large sibships are more effective than collecting more sibships or more repeats.

## Discussion

In this work, we proposed a variance component model to map candidate genes when the quantitative trait is measured repeatedly. A notable feature of our model is to accommodate a potential gene-time interaction. In the existing literature, longitudinal information on the trait is sometimes re-processed into a single trait and then the standard variance component model is applied [7]. Agreeing with other authors, we believe it is useful to have a unified model so that formal statistical inference can be performed. This benefit is evident from the simulation reported here.

We should note that the power is low with the sample sizes that we considered when the significance level is set at 0.0001. Since our purpose is to compare the power in various design settings, the absolute level of power is not critical. This is purely to reduce the computational time for our simulation. In practice, if an 80% power is desirable, for example, both the sample size and simulation replication should be increased. Despite the fact that the longitudinal study design are very popular in epidemiological and medical research, its use is still limited in linkage analysis [11]. Here, we only discuss a basic model to explore the potential of using longitudinal data and to investigate cost effective designs. Our model is related to, but has a simpler structure than that of de Andrade et al. [11]. We focus on the time at which the data are collected, but different study subjects may have data available at different time points from others. We also allow a potentially general temporal trend to interact with the genetic effect. In contrast, de Andrade et al. [11] proposed a model that assumed an individual genetic effect at every time point, which requires a uniform time schedule for all study subjects. This is a reasonable assumption for some studies including the Framingham Heart Study, but it may become restrictive to other studies.

Clearly, many important research issues warrant further investigation. For example, we need to consider gene-gene interactions, gene-environment interactions, and more general forms of gene-time interaction and fixed effects. Other classic issues including sample selection, ascertainment bias, multiple genes, and imprinting also require further investigations.

## Conclusion

We conducted a number of simulation studies to explore the increment of power when the number of sibships is increased, when the number of repeated measures is increased, and when the size of families is increased. While we expect that these factors enhance the power, how they do so is rather intriguing. Our results can provide useful guidance for designing a genetic, longitudinal study to balance the cost, feasibility, and power. For example, collecting a small number of families with a large sibship is more effective than collecting a comparatively large number of families with a small sibship. Collecting fewer families with more repeated measures may or may not lead to more power than collecting more families with fewer repeated measures, depending on the underlying genetic models. In general, however, the relationship between the power and design is subtle, and depends on the significance level and obviously the size of genetic effects. It is wise to conduct appropriate power simulations before a genetic, longitudinal study is carried out so that the cost, the feasibility, and power can be balanced. Software can be requested from the authors for such simulations.

Although our simulations were based on nuclear families, our model can handle general pedigrees as we have used it to analyze data from the Framingham Heart Study for which the pedigree size was, on average, 5 and ranged from 2 to 29.

## Methods

### The model and methods

Let *y *denote a quantitative trait. For convenience, we first consider one pedigree. By assuming-independence between pedigrees, it is straightforward to multiply the likelihood from multiple pedigrees.

Let *i *refer to the *i*th member in a pedigree and *t*_*ij *_be the time when the quantitative trait is measured at the *j*th occasion, *j *= 1,...,*T*_*i *_and *i *= 1,...,*n*. Consider the model:

*y*_*i*_(*t*_*ij*_) *= f*(*X*_*i*_, *t*_*ij*_) + *s*(*t*_*ij*_)*γ*_*i*1 _+ *γ*_*i*2 _+ *e*_*i*_(*t*_*ij*_),     (1)

where *f*(*X*_*i*_, *t*_*ij*_) is a function of the fixed effect *X*_*i *_and time *t*_*ij*_, *s*(*t*_*ij*_) a simple parametric function to accommodate time variant genetic effects, *γ*_*i*1 _the random effect for a major gene, *γ*_*i*2 _the random effect for unspecified polygenic effects over the genome, and *e*_*i*_(*t*_*ij*_) the measurement error, *j *= 1,...,*T*_*i *_and *i *= 1,...,*n*. We assume that *γ*_*i*1_, *γ*_*i*2_, and *e*_*i *_are independent, although *e*_*i*_(*t*_*ij*_), *j *= 1,...,*T*_*i*_, has a within-subject correlation structure that needs to specified on a case-by-case basis. It follows:

*cov*(*y*_*i*_(*t*), *y*_*l*_(*u*)) *= s*(*t*)*s*(*u*)*cov*(*γ*_*i*1_, *γ*_*l*1_) + *cov*(*γ*_*i*2_, *γ*_*l*2_) + *δ*_(*i *= *l*)_*σ*(*t*, *u*),

where *σ*(*t*, *u*) is the covariance function for *e*(*t*) and *e*(*u*) and *δ*_(*i *= *l*) _is the identity indicator. In addition, the covariances of *γ*_*i*1 _and *γ*_*i*2 _can be partitioned into additive and dominant variances as follows:

$\begin{array}{c} cov(\gamma_{i1},\gamma_{l1})=(k_{1,il}/2+k_{2,il})\sigma_{a1}^{2}+k_{2,il}\sigma_{d1}^{2}\\ =\pi_{il}\sigma_{a1}^{2}+k_{2,il}\sigma_{d1}^{2} \end{array}$

and

$cov(\gamma_{i2},\gamma_{l2})=2\varphi_{il}\sigma_{a2}^{2}+\tau_{il}\sigma_{d2}^{2},$

where *k*_1,*il *_and *k*_2,*il *_represent the *k *coefficients of [14] for the probability of members *i *and *l *sharing 1 and 2 alleles, respectively, identity by decent (IBD) at the locus of interest, *φ *and *τ *are respectively the expected kinship coefficient and the expected probability of sharing 2 alleles IBD over the residual components of the genome, $\sigma_{a1}^{2}$ and $\sigma_{d1}^{2}$ are respectively the additive and dominant genetic variances at the locus of interest, and $\sigma_{a2}^{2}$ and $\sigma_{d2}^{2}$ are respectively the total additive and dominant genetic variances over the residual components of the genome.

With *s*(*t*_*ij*_) = 1, without *f*(*X*_*i*_, *t*_*ij*_), and without repeated measures, model (1) reduces to the standard variance component model for quantitative traits. Thus, model (1) is an extension of the standard variance component model to accommodate the repeated measures with a structured gene-time interaction. The structured gene-time interaction distinguishes model (1) from the existing models (e.g. [11]). Although *γ*_*i*1 _does not depend on time, the manifest of genetic effects over time is accomplished through *s*(*t*). For simplicity, model (1) does not consider time-varying polygenic effects because there is no interaction term between *γ*_*i*2 _and time.

### Parameter estimation and hypothesis testing

If we arrange the phenotype in model (1) as

**y **= (*y*_1_(*t*_11_),...,$y_{1}(t_{1T_{1}})$,...,*y*_*i*_(*t*_*i*1_),...,$y_{i}(t_{iT_{i}})$,...,*y*_*n*_(*t*_*n*1_),...,$y_{n}(t_{nT_{n}})$)',     (2)

then its covariance matrix is

$\begin{array}{ccccc} \Sigma & = & \left(\begin{array}{cccc} s(t_{1}) & 0 & \cdots & 0\\ 0 & s(t_{2}) & \cdots & 0\\ \vdots & \vdots & \vdots & \vdots \\ 0 & 0 & \cdots & s(t_{n}) \end{array}\right) & \left(\Pi \sigma_{a1}^{2}+K\sigma_{d1}^{2}\right) & \left(\begin{array}{cccc} s(t_{1}{)}^{\prime } & 0 & \cdots & 0\\ 0 & s(t_{2}{)}^{\prime } & \cdots & 0\\ \vdots & \vdots & \vdots & \vdots \\ 0 & 0 & \cdots & s(t_{n}{)}^{\prime } \end{array}\right)\\ & + & \left(\begin{array}{cccc} 1_{T_{1}} & 0 & \cdots & 0\\ 0 & 1_{T_{2}} & \cdots & 0\\ \vdots & \vdots & \vdots & \vdots \\ 0 & 0 & \cdots & 1_{T_{n}} \end{array}\right) & \left(2\Phi \sigma_{a2}^{\text{2}}+\Omega \sigma_{d2}^{\text{2}}\right) & \left(\begin{array}{cccc} {1^{\prime }}_{T_{1}} & 0 & \cdots & 0\\ 0 & {1^{\prime }}_{T_{2}} & \cdots & 0\\ \vdots & \vdots & \vdots & \vdots \\ 0 & 0 & \cdots & {1^{\prime }}_{T_{n}} \end{array}\right)+E, \end{array}\;\left(3\right)$

Where *s*(*t*_*i*_) = (*s*(*t*_*i*1_),..., *s*(^t^iT_i_)', Π = (*π*_*il*_)_*n *× *n*_, *K *= (*k*_2,*il*_)_*n *× *n*_, Φ = (*φ*_*il*_)_*n *× *n*_, Ω = (*τ*_*il*_)_*n *× *n*_, $1_{T_{i}}$ is a vector of *T*_*i *_1's, and *E *is a block diagonal matrix,

$E=\left(\begin{array}{cccc} E_{1} & 0 & \cdots & 0\\ 0 & E_{2} & \cdots & 0\\ \vdots & \vdots & \vdots & \vdots \\ 0 & 0 & \cdots & E_{n} \end{array}\right),$

in which

$E_{i}=\left(\begin{array}{cccc} \sigma (t_{i1},t_{i1}) & \sigma (t_{i1},t_{i2}) & \cdots & \sigma (t_{i1},t_{iT_{i}})\\ \sigma (t_{i2},t_{i1}) & \sigma (t_{i2},t_{i2}) & \cdots & \sigma (t_{i2},t_{iT_{i}})\\ \vdots & \vdots & \vdots & \vdots \\ \sigma (t_{iT_{i}},t_{i1}) & \sigma (t_{iT_{i}},t_{i2}) & \cdots & \sigma (t_{iT_{i}},t_{iT_{i}}) \end{array}\right).$

For example, if *σ*(*t*, *u*) = *σ*^2^*e*^-*α*|*t *- *u*|^, we have

$E_{i}=\sigma^{2}\left(\begin{array}{cccc} 1 & e^{-\alpha |t_{i1}-t_{i2}|} & \cdots & e^{-\alpha |t_{i1}-t_{iT_{i}}|}\\ e^{-\alpha |t_{i2}-t_{i1}|} & 1 & \cdots & e^{-\alpha |t_{i2}-t_{iT_{i}}|}\\ \vdots & \vdots & \vdots & \vdots \\ e^{-\alpha |t_{iT_{i}}-t_{i1}|} & e^{-\alpha |t_{iT_{i}}-t_{i2}|} & \cdots & 1 \end{array}\right).$

In this work, we assume that *γ*_*i*1_, *γ*_*i*2_, and *e*_*i *_have normal distributions with mean 0. If the normality is not assumed, a generalized estimating equation approach can be adopted. However, we will not explore this approach here. For clarity, we consider a specific version of model (1). Namely, let *f*(*X*_*i*_, *t*_*ij*_)* = β*_0 _+ *t*_*ij*_*β*_1 _+ *X*_*i*_(*t*_*ij*_)*β*_2_, where *β*_2 _is a *p*-vector of parameters. In addition, assume that *s*(*t*) is a first-order polynomial function, *s*(*t*) = *s*_0 _+ *s*_1_*t*.

Let

*β *= (*β*_0_, *β*_1_, *β*_2_)'     (4)

be the vector of fixed effect parameters, and

$\theta =(\sigma_{a1}^{2},\sigma_{d1}^{2},\sigma_{a2}^{2},\sigma_{d2}^{2},s_{0},s_{1},\sigma^{2},\alpha {)}^{\prime }\;\left(5\right)$

be the vector of the covariance parameters. We estimate these parameters through the restricted maximum likelihood (REML) approach introduced by Patterson and Thompson [15] which takes into account the loss in degrees of freedom resulting from estimating fixed effects and avoids the bias in the estimation of covariance parameters.

Note that **y **has a multivariate normal distribution with mean *Aβ *and covariance Σ, where

$A=\left(\begin{array}{ccc} 1 & t_{11} & X_{1}(t_{11})\\ \vdots & \vdots & \vdots \\ 1 & t_{1T_{1}} & X_{1}(t_{1T_{1}})\\ \vdots & \vdots & \vdots \\ 1 & t_{n1} & X_{n}(t_{n1})\\ \vdots & \vdots & \vdots \\ 1 & t_{nT_{n}} & X_{n}(t_{nT_{n}}) \end{array}\right).\;\left(6\right)$

Now, let us consider *M *independent pedigrees. Let

$Y=\left(\begin{array}{c} y^{\left(1\right)}\\ y^{\left(2\right)}\\ \vdots \\ y^{\left(M\right)} \end{array}\right),A=\left(\begin{array}{c} A^{\left(1\right)}\\ A^{\left(2\right)}\\ \vdots \\ A^{\left(M\right)} \end{array}\right),\Sigma =\left(\begin{array}{cccc} \Sigma^{\left(1\right)} & 0 & \cdots & 0\\ 0 & \Sigma^{\left(2\right)} & \cdots & 0\\ \vdots & \vdots & \vdots & \vdots \\ 0 & 0 & \cdots & \Sigma^{\left(M\right)} \end{array}\right)$

where **y**^(*m*)^, *A*^(*m*) ^and Σ^(*m*) ^are of the forms (2), (6), and (3) respectively for the *m*th pedigree, *m *= 1,...,*M*.

The REML log likelihood is given by

$L(\beta ,\theta )=-\frac{1}{2}\log|\Sigma |-\frac{1}{2}\log|A^{\prime }\Sigma^{-1}A|-\frac{1}{2}(Y-A\beta {)}^{\prime }\Sigma^{-1}(Y-A\beta ).$

Maximizing *L*(*β*, *θ*) with respect to *β *gives

$\hat{\beta }$ = (*A*'Σ^-1^*A*)^-1 ^*A*'Σ^-1^*Y*.

Plugging $\hat{\beta }$ into the log likelihood, we have

$\begin{array}{l} \;\;\;\;\;l(\theta )=L(\hat{\beta },\theta )=-\frac{1}{2}\log|\Sigma |-\frac{1}{2}\log|A^{\prime }\Sigma^{-1}A|-\frac{1}{2}(Y-A\hat{\beta }{)}^{\prime }\Sigma^{-1}(Y-A\hat{\beta })\\ =-\frac{1}{2}\log|\Sigma |-\frac{1}{2}\log|A^{\prime }\Sigma^{-1}A|-\frac{1}{2}Y^{\prime }\Sigma^{-1}(I-A{\left(A^{\prime }\Sigma^{-1}A\right)}^{-1}A^{\prime }\Sigma^{-1})Y\\ =-\frac{1}{2}\log|\Sigma |-\frac{1}{2}\log|A^{\prime }\Sigma^{-1}A|-\frac{1}{2}Y^{\prime }PY. \end{array}$

where *P *= Σ^-1^(*I *- *A*(*A*'Σ^-1^*A*)^-1^*A*'Σ^-1^). The REML estimator for *θ *is obtained by maximizing the log-likelihood *l*(*θ*). Substituting the estimator for *θ *into $\hat{\beta }$ gives the REML estimator for *β*.

Based on the theory on matrix derivatives, we have $\frac{\partial \log|\Sigma |}{\partial \theta }=tr\left(\Sigma^{-1}\frac{\partial \Sigma }{\partial \theta }\right),\frac{\partial \Sigma^{-1}}{\partial \theta }=-\Sigma^{-1}\frac{\partial \Sigma }{\partial \theta }\Sigma^{-1}$, $\frac{\partial \log|A^{\prime }\Sigma^{-1}A|}{\partial \theta }=-tr({\left(A^{\prime }\Sigma^{-1}A\right)}^{-1}A^{\prime }\Sigma^{-1}\frac{\partial \Sigma }{\partial \theta }\Sigma^{-1}A)$ and $\frac{\partial P}{\partial \theta }=-P\frac{\partial \Sigma }{\partial \theta }P$. Therefore, the first-order partial derivative of the log likelihood *l*(*θ*) with respect to *θ *gives

$\begin{array}{c} \frac{\partial l(\theta )}{\partial \theta }=-\frac{1}{2}tr(\Sigma^{-1}\frac{\partial \Sigma }{\partial \theta })+\frac{1}{2}tr({\left(A^{\prime }\Sigma^{-1}A\right)}^{-1}A^{\prime }\Sigma^{-1}\frac{\partial \Sigma }{\partial \theta }\Sigma^{-1}A)+\frac{1}{2}Y^{\prime }P\frac{\partial \Sigma }{\partial \theta }PY\\ =-\frac{1}{2}tr(P\frac{\partial \Sigma }{\partial \theta })+\frac{1}{2}Y^{\prime }P\frac{\partial \Sigma }{\partial \theta }PY, \end{array}$

and the second-order partial derivative of the log likelihood *l*(*θ*) with respect to *θ *gives

$\frac{\partial^{2}l(\theta )}{\partial \theta^{\text{2}}}=-\frac{1}{2}tr(-P\frac{\partial \Sigma }{\partial \theta }P\frac{\partial \Sigma }{\partial \theta }+P\frac{\partial^{2}\Sigma }{\partial \theta^{\text{2}}})-\frac{1}{2}Y^{\prime }(2P\frac{\partial \Sigma }{\partial \theta }P\frac{\partial \Sigma }{\partial \theta }-P\frac{\partial^{2}\Sigma }{\partial \theta^{\text{2}}})PY.$

Denote the matrix of the negative second partial derivatives of *l*(*θ*) as

$g(\theta )=-\frac{\partial^{2}l(\theta )}{\partial \theta^{2}}.$

A Newton-Raphson algorithm yields

${\hat{\theta }}_{\left(n+1\right)}={\hat{\theta }}_{\left(n\right)}+g^{-1}({\hat{\theta }}_{\left(n\right)})\frac{\partial l({\hat{\theta }}_{\left(n\right)})}{\partial \theta }.$

Iterate until changes in successive estimates of all parameters are sufficiently small. Let $\hat{\theta }$ be the converged estimate of *θ*.

If (β_*_, θ_*_) is the vector of true parameter values, based on classical statistical theory, ($\hat{\beta }$ - β_*_, $\hat{\theta }$ - θ_*_) follows asymptotically a multivariate normal distribution with mean 0. And the asymptotical covariance matrix can be estimated by *I*^-1^($\hat{\beta }$, $\hat{\theta }$), where *I*(*β*, *θ*) is the information matrix.

Linkage is tested by a likelihood ratio test by comparing the likelihood under the alternative hypothesis in which the genetic variance component due to the testing QTL is estimated with that under the null hypothesis of the genetic variance due to the testing QTL being equal to zero (no linkage). Twice the natural logarithm of the likelihood ratio of these two models may have a complex asymptotic distribution of a mixture of *χ*^2 ^distributions [16] and what kind of asymptotic distribution depends on how *s*(*t*) is defined.

## Authors' contributions

HZ contributed to the conception and design of the study, analysis and interpretation of data, and XZ contributed to the design of the study, wrote the programs, and performed the simulation analysis. Both authors have been involved in writing the manuscript and approved this final version.
